# Enhanced Solar Efficiency via Incorporation of Plasmonic Gold Nanostructures in a Titanium Oxide/Eosin Y Dye-Sensitized Solar Cell

**DOI:** 10.3390/nano12101715

**Published:** 2022-05-17

**Authors:** Sanele Nyembe, Francis Chindeka, Gebhu Ndlovu, Andile Mkhohlakali, Tebello Nyokong, Lucky Sikhwivhilu

**Affiliations:** 1 Analytical Chemistry Division/Mintek, Private Bag X3015, Randburg, Johannesburg 2125, South Africa; andilem@mintek.co.za; 2 Institute for Nanotechnology Innovation, Department of Chemistry, Rhodes University, Grahamstown 6140, South Africa; f.chindeka@ru.ac.za (F.C.); t.nyokong@ru.ac.za (T.N.); 3 DST/Mintek Nanotechnology Innovation Centre, Mintek, Private Bag X3015, Randburg, Johannesburg 2125, South Africa; gebhun@mintek.co.za (G.N.); luckys@mintek.co.za (L.S.); 4 Department of Chemistry, Faculty of Science, Engineering and Agriculture, University of Venda, Private Bag X5050, Thohoyandou 0950, South Africa

**Keywords:** gold nanoparticles, titanium oxide, dye-sensitized solar cell, photon-to-electron conversion efficiency, hot electron injection mechanism, charge recombination

## Abstract

Plasmonic gold nanoparticles significantly improved the efficiency of a TiO_2_ and Eosin Y based dye-sensitized solar cell from 2.4 to 6.43%. The gold nanoparticles’ sizes that were tested were 14 nm, 30 nm and 40 nm synthesized via the systematic reduction of citrate concentration using the Turkevich method. Prestine TiO_2_ without plasmonic gold nanoparticles yielded an efficiency of 2.4%. However, the loading of 40 nm gold nanoparticles into the TiO_2_ matrix yielded the highest DSSC efficiency of 6.43% compared to 30 nm (5.91%) and 14 nm (2.6%). The relatively high efficiency demonstrated by plasmonic gold nanoparticles is ascribed to light absorption/scattering, hot electron injection and plasmon-induced resonance energy transfer (PIRET), influenced by the size of the gold nanoparticles.

## 1. Introduction

Global electrical energy consumption is currently at an overwhelming 16 terawatt (TW), which includes transportation, agriculture, industry, heating, etc. [1,2]. The current global population increase rate of 1.1% per year and the adaption of the modern way of life due to urbanization will catapult the global energy consumption to 29 TW in less than 15 years [3,4]. The majority of the 16 TW energy consumption (12 TW) is from burning fossil fuels [3]. Hence, there is a growing requirement for global renewable energy sources in order to eradicate the dependency on finite fossil fuels. Dye-sensitized solar cells (DSSCs) are a promising renewable energy source owing to the low production cost and relatively high photo-to-electron conversion efficiency [5,6]. The highest efficiency of 12.3% was reported by Gratzel et al. and ever since then efforts to enhance DSSC efficiency have been attempted, with no success so far [7]. High efficiencies have been reported for perovskite-based solar cells; however, the cells show poor stability [8], limiting their commercialization. Hence, there is a need for stable materials for DSSCs with relatively higher efficiency. DSSCs are based on a dye that captures photons from the incident light promoting electrons from valence into a conduction band. The promoted electrons from the dye are then transferred into the conduction band of metal oxide-based semiconductor materials such as titanium oxide and zinc oxide [7,9,10]. The most common challenges with DSSCs are: (i) dye insufficient light absorption; (ii) high charge recombination rates on both the metal oxide semiconductor and the dye; and lastly, the (iii) insufficient charge separation of the metal oxide semiconductor [5,11,12].

The most used metal oxide semiconductor for DSSCs is titanium oxide because it is stable and relatively cheap. However, it absorbs the ultraviolet light of the light spectrum and has a 3.2 eV wide band-gap leading to low photon to electron conversion efficiency [7]. To improve light absorption of the titanium oxide semiconductor in the visible region, metal nanostructures such as copper, silver, gold and aluminum have been used to improve light absorption across the solar spectrum [13,14,15,16,17]. Furthermore, the interest in metal nanostructures for DSSCs is based on trapping photons efficiently based on localized surface plasmon resonance (LSPR). This is a phenomenon of trapping electron cascades into three mechanisms that are crucial in DSSC, namely: (i) hot electron injection, (ii) incident light absorption/scattering and (iii) plasmon-induced resonance energy transfer [18,19]. These mechanisms increase the influx of excited electrons into the semiconductor materials that are affected by the size, shape and stabilizing agent of the metal nanoparticles [19,20]. The increase in DSSC efficiency has been reported with an increase in the size of plasmonic Ag and AuNPs, with increments of around 13% and 28%, respectively [21,22]. In this study, we used gold nanoparticles (AuNPs) with different sizes to make Au/TiO_2_ nanocomposites for DSSCs. We then studied the effects of gold nanoparticles’ physical properties such as size and shape on the efficiency of the DSSC. In this work, we show that increasing the size of the AuNPs improves the efficiency of the DSSC with increments that are higher than have been reported before [21,22].

## 2. Materials and Methods

Titanium oxide (P25), tri-sodium citrate, tetrachloroaurate, indium doped tin oxide (ITO) glass slides, iodine, lithium iodide, Eosin Y dye and tetraisopropoxide were all purchased from Sigma Aldrich, Johannesburg, South Africa. The physical properties of the samples were examined by TEM, JEOL operated at 200 kV. The X-ray diffractometer (Bruker D8 Discover, Billerica, MA, USA), equipped with a copper radiation source (1.5405 °A), was used to analyze the material crystalline phases. The optical properties were investigated using a Shimadzu^®^ UV-2550 spectrophotometer (Kyoto, Japan). The performance of the DSSC was measured using a Metrohm potentiostat (PGSTAT302N, Herisau, Switzerland) coupled with an LED driver kit using warm white light.

### 2.1. Synthesis of Gold Nanoparticles (AuNPs) with Different Sizes

Spherical AuNPs were prepared following a Turkevich method [23] via an in-situ nucleation method by mixing citrate and gold salt with various concentrations. Masses of tri-sodium citrate of 192.10, 96.10 and 19.20 mg dissolved in water were added into a solution of tetrachloroaurate (mass of 75.83 mg dissolved in water) to achieve mass ratios of 0.7, 0.5 and 0.2 of citrate to tetrachloroaurate. The samples were heated to a boiling point while being stirred then cooled to an ambient temperature. The samples were characterized after isolation in the dark cupboard for 24 h. The resulting AuNPs have TEM sizes of 14, 30 and 40 nm, for mass ratios of 0.7, 0.5 and 0.2 of citrate to tetrachloroaurate, respectively, and will be represented as Au-14 nm, Au-30 nm and Au-40 nm throughout the manuscript.

### 2.2. Synthesis of Au/TiO_2_ Nanocomposite

The Au/TiO_2_ nanocomposites (2.5 wt.%) were prepared by freeze drying the AuNPs solutions to form AuNPs’ nanopowders, that were then mixed with TiO_2_ powder in ethanol, separately for each nanoparticle size. Each solution was ultrasonicated for 5 min and vigorously shaken using an electric shaker for 1 h at room temperature. The Au/TiO_2_ nanocomposites were centrifuged at 4000 rpm to recover the Au/TiO_2_ nanocomposites. Finally, the samples were dried in the oven at 80 °C for overnight.

### 2.3. Fabrication of Au/TiO_2_ Customized Photoanode

A mass of 100 mg of Au-TiO_2_ nanocomposite, 2 mL of ethanol and a drop of tetraisopropoxide solution were all mixed to form a paste. The paste was stirred and ultrasonicated for 5 min each to form a homogeneous mixture. The Au/TiO_2_ nanocomposite paste was coated onto the photoanode (indium tin oxide (ITO) glass) using a blade [22]. The coating was dried in air for 10 min and partially sintered using a hotplate at 150 °C. The samples were then left to cool in air to ambient temperature. Au/TiO_2_ thin film was measured to be ≈40 µm for all the samples.

### 2.4. Fabrication of Dye-Sensitized Solar Cell and Testing

**Figure 1:**
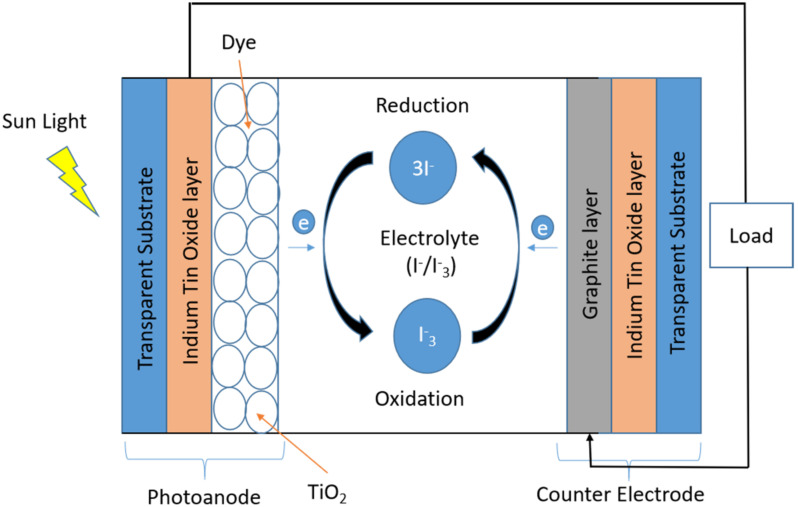
A dye-sensitized solar cell schematic diagram [24].

The Au/TiO_2_ coated ITO glass (photoanode) was immersed in 0.6 mM Eosin Y dye solution for a duration of 24 h at ambient temperature. The photoanode was rinsed with ethanol and gently wiped to remove access dye. A thin parafilm with an opening of 0.6 cm × 1.5 cm was placed onto the dye adsorbed photoanode to achieve a 0.9 cm^2^ active area for the cell performance analysis. A redox electrolyte (iodine/lithium iodide) solution was introduced into the cell assembly through the active area. A graphite-coated ITO was clamped together with the photoanode with the parafilm in the middle to complete the DSSCs that were tested (Figure 1).

## 3. Results

**Figure 2:**
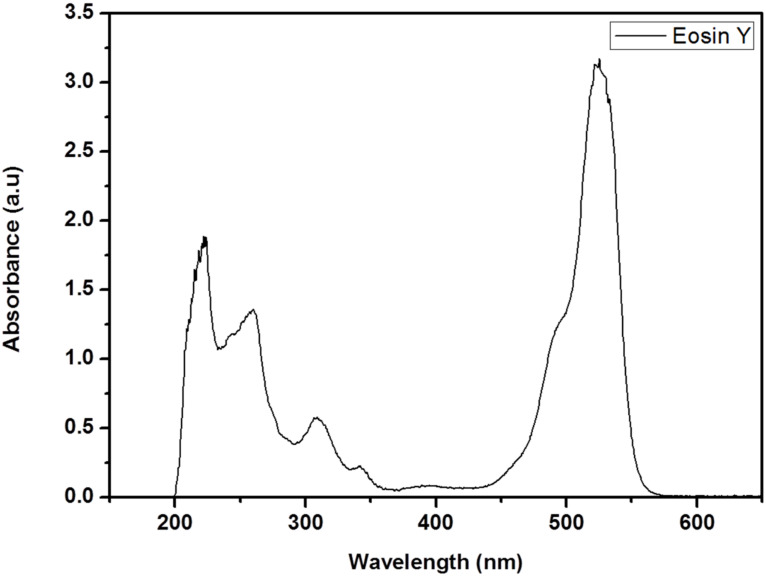
The ultraviolet-visible spectrum of Eosin Y dye suspended in dimethyl sulfoxide solvent.

Figure 2 shows the UV-Vis spectrum of Eosin Y with five distinct peaks at wavelengths of 230, 260, 310, 340 and 523 nm. The most intense peak of Eosin Y at a wavelength of 523 nm absorbed visible light (350 to 700 nm) which is important for the DSSC application.

**Figure 3:**
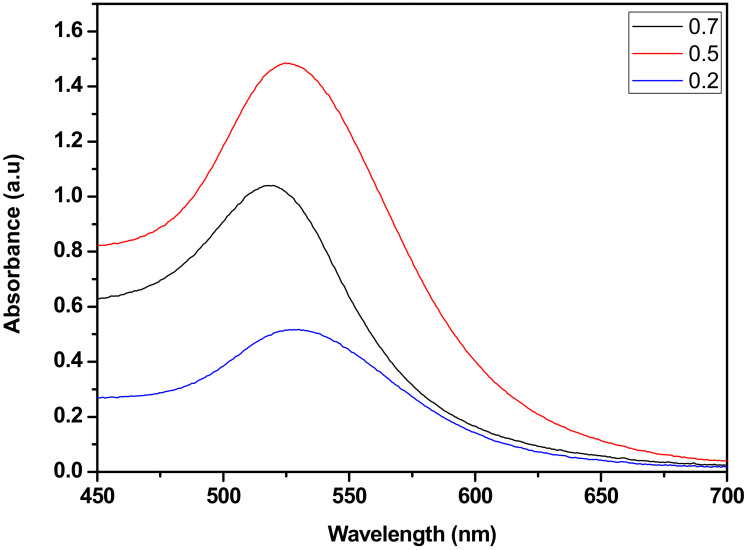
The UV-Vis spectra of the samples with various citrate/metal precursor ratios.

Figure 3 shows the UV-Vis spectra of samples (Au-40 nm (0.2), Au-30 nm (0.5) and Au-14 nm (0.7), respectively). The UV-Vis spectra of the samples revealed a monomodal peak at the wavelength positions of 528, 527 and 518 nm. A monomodal peak of the samples implies that the nanoparticles were monodispered. The wavelength position of the UV-Vis was a typical surface plasmon resonance (SPR) band of gold nanostructures in solution [25]. There was a red-shift when the citrate concentration decreased which suggests an increase in the particle size from 0.7 to 0.2 ratio as confirmed by UV-Vis in Figure 3. 

**Figure 4:**
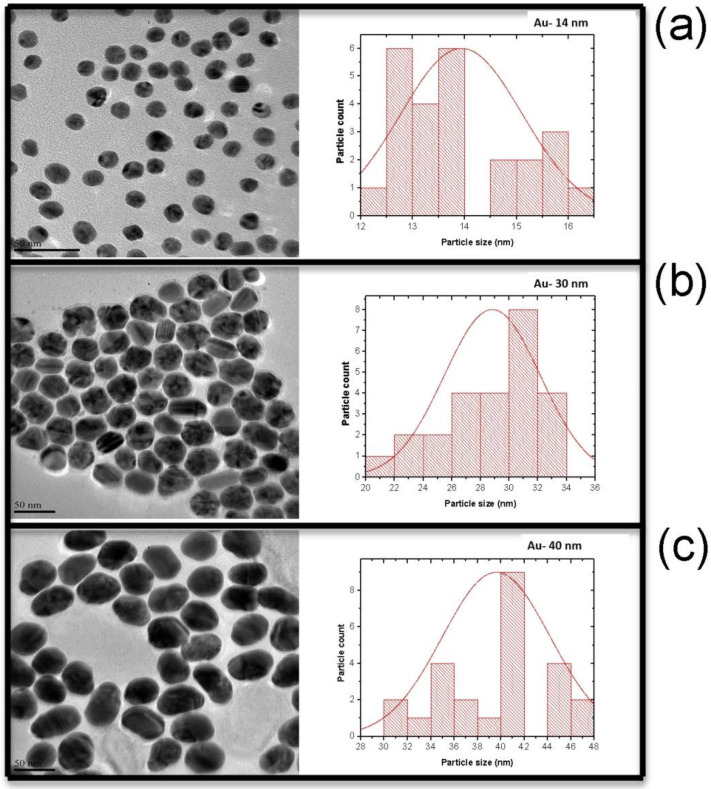
The transmission electron microscope images and size distribution histograms of AuNP samples synthesized with (**a**) 0.7, (**b**) 0.5 and (**c**) 0.2 citrate salt/gold salt ratio.

AuNPs were synthesized by the classic Turkevich method with varying citrate/gold salt ratios [26]. Figure 4 shows TEM images of the various samples (ratios: 0.7, 0.5 and 0.2) with their respective size distribution histograms. The TEM images revealed that the samples had particle sizes of 14 (0.7), 30 (0.5) and 40 nm (0.2). The TEM showed monodispered AuNPs in line with the single peak observed from the UV-Vis spectrum. Interestingly, the shape of the AuNPs became increasingly irregular with a decrease in the citrate/gold salt ratio. A limited concentration of citrate molecules resulted in a formation of an oval shape and/or other irregular structures (Figure 4).

The results show that a 0.7 citrate ratio to gold salt led to the formation of small AuNPs (14 nm). As the citrate concentration was systematically reduced, the size of the nanoparticles increased to 30 nm and then to 40 nm. It is noteworthy that the reduction of citrate concentration implies an increase in gold ion concentration. An equilibrium of citrate ions and gold ions is important in determining the morphology of gold nanoparticles. The citrate moiety hinders the growth of gold crystal planes, which control the size of gold nanoparticles.

### 3.1. Physical and Structural Properties of TiO_2_ Nanoparticles

**Figure 5:**
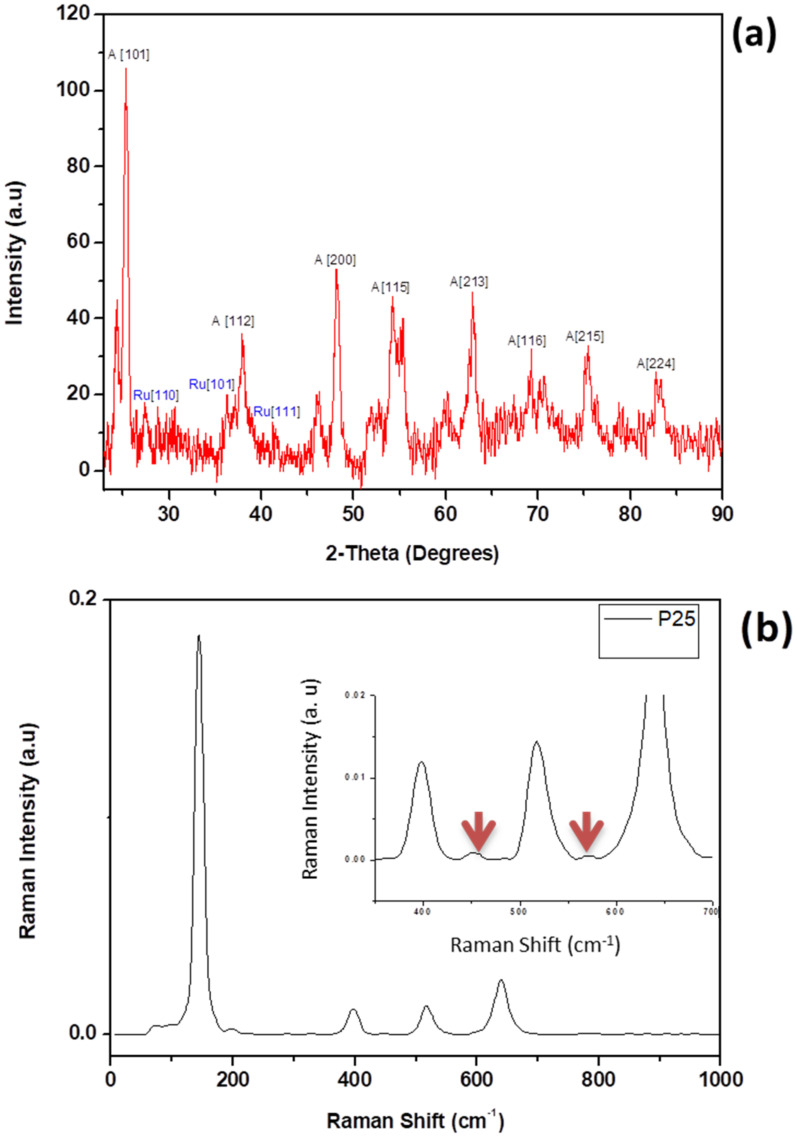
The (**a**) XRD and (**b**) Raman spectra of titanium oxide nanoparticles with the arrows showing the rutile phase.

Figure 5a shows the XRD pattern of commercial TiO_2_ (P25) nanopowder. The XRD pattern revealed narrow peaks implying a crystalline sample. Peaks at 25° {101}, 36° {112}, 48° {200}, 54° {115}, 63° {213}, 69° {116}, 76° {215} and 84° {224} 2-Theta values were all indexed to the anatase phase and the peaks at 2-Theta values of 28° {110} and 36° {101} were indexed to the rutile phase. These results show that the TiO_2_ powder was a blend of anatase and rutile phases (80%/20%) (JCPDS, Card No. 4-784). Figure 5b shows the Raman spectrum of the TiO_2_ nanoparticles. The Raman spectrum revealed typical anatase structure bands (Eg (142 cm^−1^), B1g (399 cm^−1^), A1g (520 cm^−1^) and Eg (641 cm^−1^) [27]. Figure 4b (insert) showed A1g (452 cm^−1^) and Eg (570 cm^−1^) bands which are characteristic of the rutile phase [27]. The XRD and Raman results show that the anatase phase was a predominant phase in the samples. 

**Figure 6:**
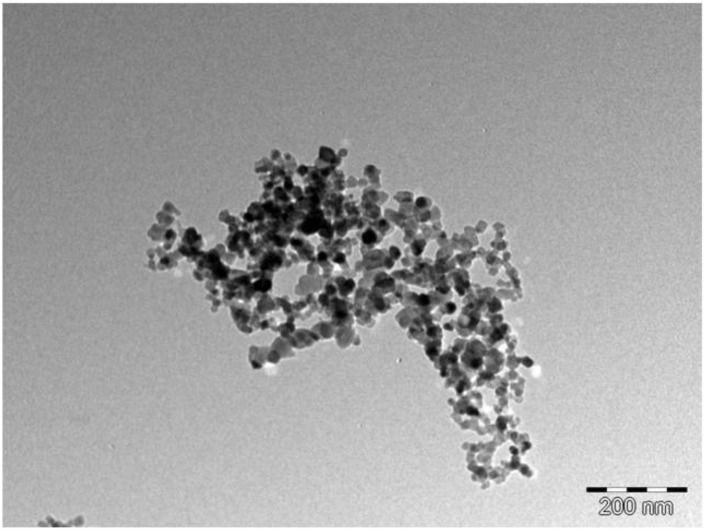
TEM image of TiO_2_ nanoparticles.

The physical properties of TiO_2_ were studied by TEM and the image is shown in Figure 6. A TEM analysis revealed that TiO_2_ nanoparticles were in a spherical form, having a diameter of 20 nm (range of 16 to 28 nm). The observed relatively uniform TiO_2_ nanoparticles are indicative of the monodispersive nature of the nanoparticles. The observed relatively narrow particle size distribution of TiO_2_ suggests a very high surface area and enhanced adsorption binding sites for dye for the DSSC application. The TiO_2_ nanoparticles appeared to have a monolithic structure and appeared to be stacked on top of each other, making other particles appear darker than the others.

### 3.2. Physical and Structural Properties of Au/TiO_2_ Nanocomposite

**Figure 7:**
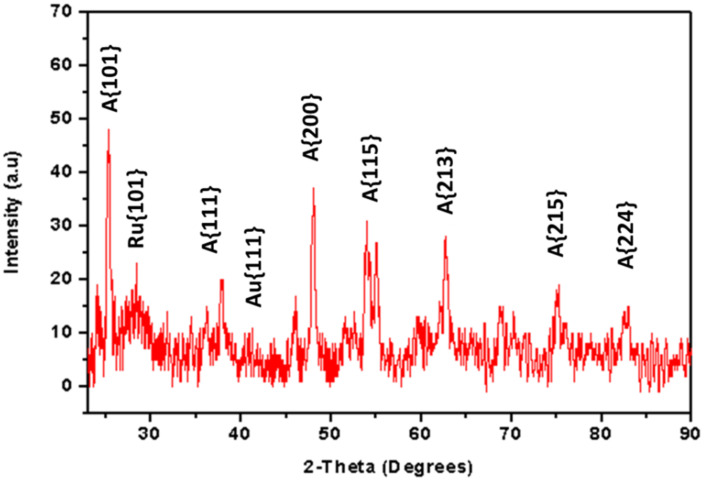
XRD pattern of Au/TiO_2_ nanocomposite (0.7 ratio).

The structural properties of the Au/TiO_2_ nanocomposite (from 0.7 ratio) were characterized using XRD in Figure 7. The XRD spectrum revealed peaks at 25° {101}, 36° {112}, 48° {200}, 54° {115}, 63° {213}, 69° {116}, 76° {215} and 84° {224} 2-Theta angles. The peaks were all indexed to the anatase phase of TiO_2_. The peaks at 2-Theta angles of 28° {110} and 36° {101} were all indexed to the rutile phase, similar to the pristine TiO_2_ results (Figure 5). However, the peaks at 2-Theta angles of 38° {111} and 42° {200} were indexed to the gold nanoparticles, confirming the Au/TiO_2_ nanocomposite formation. Furthermore, the Au/TiO_2_ composite color changed to purple, similar to the color of gold nanoparticles in solution. 

**Figure 8:**
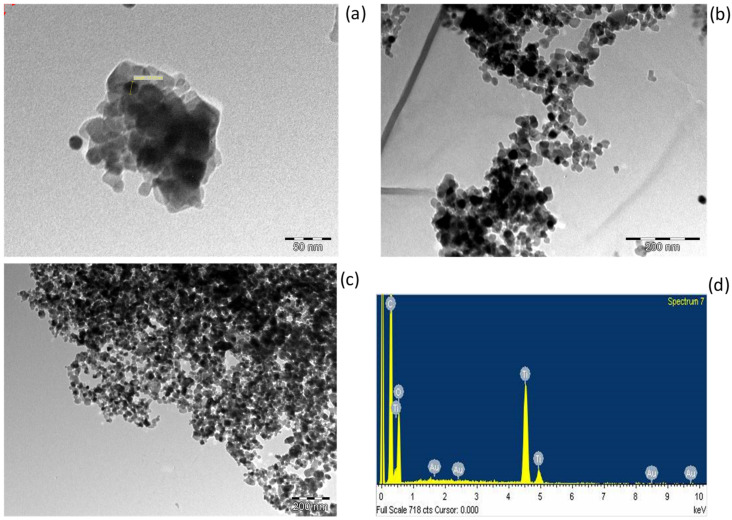
TEM image of Au/TiO_2_ nanocomposite with gold nanoparticles’ TEM sizes of (**a**) 14, (**b**) 30 and (**c**) 40 manometers with their (**d**) EDX spectrum.

Figure 8 shows the TEM images of Au-TiO_2_ nanocomposite samples incorporated with AuNP sizes of (a) 14, (b) 30 and (c) 40 nm with their respective (d) EDX spectrum. The AuNPs were identified by both their smaller size within the TiO_2_ nanoparticle matrix and by the EDX spectrum. The EDX spectrum showed Au (2%), Ti (42%), O (20%) and C (36%) elements; the carbon element was from the TEM sample holder. The scattered electrons from the TEM were directly proportional to the atomic number of the element. Au has a higher atomic number than titanium and oxygen; hence, it appeared brighter on the TEM image. This study revealed that it is a challenge to fully load the Au into the TiO_2_ nanoparticle matrix, as it can be observed that some AuNPs were found outside of the TiO_2_ nanoparticle matrix. 

### 3.3. The Plasmonic Gold Nanopaticles Induced Effects on the Solar Conversion Efficiency

**Figure 9:**
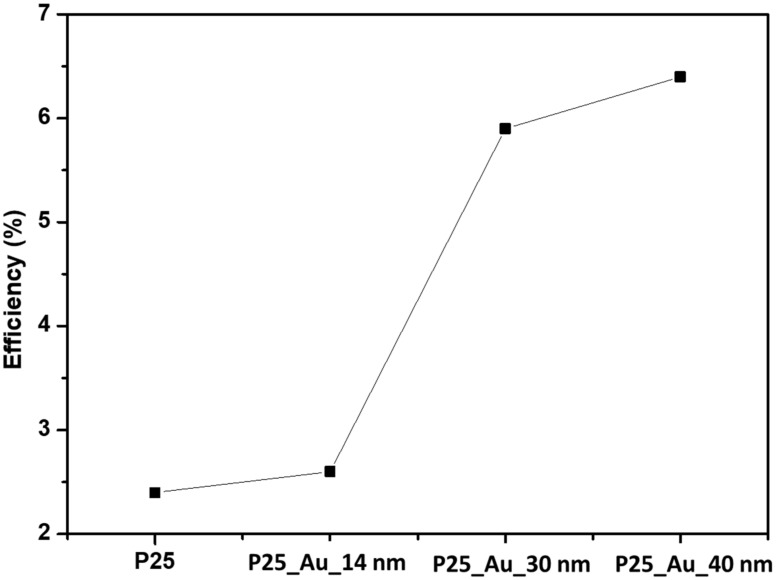
Solar cell efficiency curve comparison of TiO_2_ and different sizes of gold nanoparticles.

The efficiencies of TiO_2_ and the Au/TiO_2_ nanocomposites loaded with different sizes of AuNPs are shown in Figure 9. The results reveal that the addition of AuNPs with the size of 14 nm increased the efficiency by 8% (2.4 to 2.6%). Titanium oxide (Degussa-P25) absorbs the ultraviolet light leading to low photon-to-electron energy transition [26,28]. The increase in the efficiency was attributed to visible region light absorption (wavelength: 350 nm to 700 nm), in addition to absorption by Eosin Y dye, that was enhanced by the introduction of the plasmonic gold nanoparticles into the TiO_2_ matrix. Furthermore, Figure 9 shows an increase in solar efficiency from 2.6 to 6.4% as a direct consequence of the gold nanoparticles’ size from 14 to 40 nm. 

This observed increase, when the size of the AuNPs increased, was attributed to three mechanisms: (i) hot electron injection, (ii) light scattering/absorption and (iii) plasmon-induced resonance energy transfer. The incorporation of metal nanoparticles such as gold into the TiO_2_ semiconductor creates a metal-semiconductor hetero-junction limiting energy transfer due to the Schottky barriers emanating from the difference in Au-TiO_2_ work functions [29,30]. However, the metal nanoparticles can scatter the incident light to penetrate the semiconductor, leading to an influx of photons into the semiconductor. Furthermore, light scattered by the AuNPs promotes light absorption and charge separation in the TiO_2_, leading to better electron diffusion. The light scattering/absorption mechanism was affected by the size, shape and dielectric properties the solvent used for dispersion. Light scattered by the AuNPs can be quantified by Equation (1) [31].

(1) $$C_{\mathrm{sca}}=\frac{8}{3}{\pi k}^{4}r^{6}{\left|\frac{\varepsilon -\varepsilon m}{\varepsilon +2\varepsilon m}\right|}^{2}$$

where k = 2π/λ, λ is the light wavelength, r is the radius of gold nanoparticles, ε is the permittivity for the nanoparticles and εm is the permittivity for the surrounding medium. According to Equation (1), it is clearly demonstrated that scattered light largely depended on the radius of the gold nanoparticles, because all the AuNPs were synthesized in water with the same dielectric properties. This partly explains the high efficiency shown by DSSC with 40 nm gold nanoparticles. 

Due to differences in the work functions of gold and TiO_2_, electrons cannot freely pass through the Schottky energy barrier in the Au-TiO_2_ hetero-junction. However, after the gold nanoparticles’ absorption and scatter of light, this creates “hot electrons” within the gold nanoparticle achieved by thermal excitation. These electrons possess sufficient energy to subdue the Schottky energy barrier of the Au-TiO_2_ interface, resulting in them being injected directly into the conduction band of TiO_2_ [29]. This increases the electron influx of the TiO_2_ conduction band. The TiO_2_ conduction band does not only receive electrons from the Eosin Y dye, but it receives additional electrons from the AuNPs. Since the absorption and scattering of light is favored by larger nanostructures, this explains the high efficiency of DSSC loaded with 40 nm AuNPs compared 14 nm. Furthermore, the “hot electron injection” mechanism is affected by the proximity between the metal nanostructures and metal oxide semiconductor, affected by the stabilizing agent. The gold nanoparticles were all stabilized with citrate moiety, which was used to control the gold nanoparticles’ size. Nonetheless, the presence of the stabilizer on the surface of the gold nanoparticle could have adverse effects by limiting the accessibility of the gold surface plasmons [22]. The concentration of citrate was systematically reduced to synthesize bigger nanoparticles. This means that gold nanoparticles with 40 nm had less citrate moiety on their surface compared to 14 nm. This shows that gold nanoparticles with the size of 40 nm were in closer proximity to TiO_2_ nanoparticles compared to 14 nm AuNPs. Since the hot electron injection mechanism was affected by the distance between the metal and semiconductor, it is clear that more electron influx to the TiO_2_ conduction band was experienced for 40 nm gold nanoparticles.

Lastly, the plasmon-induced resonance energy transfer (PIRET) affects the formation of the dipole–dipole interaction formed by the excitation of the AuNPs [29]. The light-induced gold nanoparticles’ dipole–dipole pairs generate electron pairs in the semiconductor which reduces charge recombination in the TiO_2_ [28,29]. The PIRET mechanism is also affected by the metal-semiconductor proximity (contact angle) which is favored by the bigger AuNPs (40 nm). 

The solar cell efficiency was calculated according to Equation (2).

(2) $$\eta =\frac{\mathrm{ff}\;\mathrm{Voc}\;\mathrm{Jsc}}{\mathrm{Psolar}}$$

where Jsc, Voc, FF and η stand for current density, open current voltage, fill factor and solar efficiency, respectively. Results show that the current density and efficiency increased with the size of the gold nanostructures. The relatively high short circuit voltage (Jsc) was due to the optimization done to the DSSC, which was achieved by (i) enhancing the exposure area of the cell (ii) using a high energy light source with an appropriate incident light wavelength. The high currently density shown by the DSSC with 40 nm gold nanoparticles was in agreement with the high influx of electrons to the conduction band of TiO_2_, suggested by light scattering, hot electron injection and PIRET mechanisms. Table 1 show the other photovoltaic parameters of the TiO_2_ semiconductor and the different nanocomposites.

## 4. Conclusions

The gold nanoparticles’ size was controlled by the citrate concentration. Increasing the concentration of the citrate resulted in the formation of smaller AuNPs. Larger gold nanoparticles exhibited higher DSSC efficiency compared to smaller ones. The high efficiency emanating from larger AuNPs was due to light absorption/scattering, hot electron injection and a plasmon-induced resonance energy transfer mechanism. The relatively larger particles absorb and scatter light better than smaller particles, leading to a higher electron influx into the TiO_2_, which leads to higher DSSC efficiency. Larger gold nanoparticles showed a higher current density compared to smaller gold nanoparticles due to a high electron influx to the TiO_2_ caused by larger AuNPs.

## Figures and Tables

**Table 1:** Photovoltaic Parameters of DSSC based on Various Samples.

Sample Name	Jsc (mA cm^−2^)	Voc (V)	FF	η (%)
P25	1.75	0.23	0.29	2.41
TiO_2_-P25//Au-14 nm	4.36	0.24	0.30	2.60
TiO_2_-P25//Au-30 nm	26	0.29	0.30	5.91
TiO_2_-P25//Au-40 nm	45	0.08	0.23	6.43

## Data Availability

The data presented in this study are available on request from the corresponding author.
